# Preoperative risk stratification for recurrence after gross total resection of non-functioning pituitary macroadenomas: The role of tumor volume and 3D anatomical complexity — A retrospective single-center cohort study

**DOI:** 10.1016/j.bas.2026.106277

**Published:** 2026-08-07

**Authors:** V.M. Eisenkolb, P. Schneider, C. Negwer, B. Meyer, V.M. Butenschoen

**Affiliations:** Department of Neurosurgery, TUM Klinikum, Technical University of Munich, Munich, Germany

**Keywords:** Non-functioning pituitary macroadenoma, Tumor recurrence, Preoperative risk stratification, Personalized follow-up strategy

## Abstract

**Introduction:**

Recurrence after radiologically confirmed gross total resection (GTR) of non-functioning pituitary macroadenomas (NFPMAs) remains a relevant clinical challenge.

**Research question:**

Which preoperative and perioperative factors are associated with recurrence after radiological GTR of NFPMAs?

**Material and methods:**

We retrospectively analyzed 212 patients who underwent MRI-guided transnasal transsphenoidal surgery for histologically confirmed NFPMAs between 2007 and 2023 at a single tertiary neurosurgical center. Among these, 94 patients were classified as having radiological GTR based on postoperative MRI and longitudinal interdisciplinary review; 62 showed no recurrence and 32 developed recurrence during follow-up. Demographic variables, tumor configuration, suprasellar and cavernous sinus extension, surgical characteristics, histopathology, volumetric measurements, and pre-/postoperative cortisol levels were evaluated. Tumor volumes were manually segmented. Statistical comparisons included Mann–Whitney U tests, chi-squared tests, and ROC analysis.

**Results:**

Patients in the recurrence subgroup were significantly younger (mean age, 51.2 vs. 57.1 years; p = 0.008) and had significantly larger preoperative tumor volumes (10.45 cm^3^ vs. 5.54 cm^3^; p = 0.022), higher Hardy grades (p < 0.001), higher Knosp grades (p = 0.009), and more frequent suprasellar extension (90.6% vs. 64.5%; p = 0.014). ROC analysis identified a preoperative volume cutoff of 9.22 cm^3^ (AUC 0.667). Surgical approach, histological subtype, and preoperative visual field deficits were not significantly associated with recurrence. Postoperative cortisol changes differed between groups (p = 0.035).

**Discussion and conclusion:**

Larger tumor volume and more invasive radiological configuration were associated with recurrence despite radiological GTR. These parameters may help refine postoperative surveillance strategies. Prospective validation is warranted.

## Highlights

• Larger preoperative tumor volume was associated with recurrence after GTR.
• Higher Hardy and Knosp grades were linked to postoperative recurrence.
• Suprasellar extension was more frequent in tumors that later recurred.
• Histological subtype was not significantly associated with recurrence.
• Volumetry may support individualized postoperative MRI surveillance.

## 1 Introduction

Pituitary adenomas are neoplasms originating from adenohypophyseal cells and represent the second most common primary brain tumors in adults (Laws et al., 2019), with an estimated prevalence of 0.5–1 per 1000 individuals (Daly et al., 2006; Raappana et al., 2010; Fernandez et al., 2010; Tjornstrand et al., 2014). These tumors are broadly classified into microadenomas (<10 mm) and macroadenomas (≥10 mm). Additionally, they are categorized into functioning adenomas, which excessively produce pituitary hormones, and non-functioning adenomas, which do not exhibit hormone overproduction.

Non-functioning macroadenomas account for approximately 30% of all pituitary adenomas (Molitch, 2017). When not discovered incidentally, they typically become symptomatic due to their mass effect, resulting in the compression of adjacent structures. The most common clinical manifestations include visual field defects, such as bitemporal hemianopia, caused by compression of the optic chiasm (Butenschoen et al., 2021), and hormonal dysfunction (Chanson et al., 2015), resulting from pituitary stalk compression or glandular insufficiency.

The gold-standard treatment for symptomatic non-functioning pituitary macroadenomas is transnasal, transsphenoidal resection, performed using either an endoscopic or microscopic approach. The primary treatment goal is not only symptomatic relief through decompression but also long-term tumor control. Achieving gross total resection (GTR) is a crucial factor in minimizing recurrence risk. However, despite advancements in diagnostic imaging and surgical techniques, complete tumor resection remains challenging and is only achieved in approximately 60% of cases (Yu et al., 2018). Several factors influence resection success, including tumor invasion into the cavernous sinus (Pontes et al., 2023), the tumor's three-dimensional structure, and individual anatomical variations.

Given the variability in surgical outcomes and recurrence risk, identifying predictive factors for tumor regrowth after gross total resection is essential.

In this retrospective, single-center cohort study, we analyzed routinely collected clinical and radiological parameters to determine potential predictors of recurrence. By identifying high-risk patients early, we aim to contribute to a more individualized approach in follow-up management and adjuvant treatment decisions.

## 2 Material and methods

We conducted a retrospective analysis of all patients who underwent MRI-guided, transnasal transsphenoidal surgery for histologically confirmed non-functioning pituitary macroadenomas between January 2007 and December 2023 at our tertiary neurosurgical center. Parts of the overall institutional cohort have previously been analyzed with respect to progression of postoperative residual disease after STR (Eisenkolb et al., 2026). The present study addresses a distinct primary endpoint and restricts the primary analysis to patients with radiologically confirmed GTR, comparing those with and without subsequent recurrence. Histopathological reports and pre- and postoperative imaging studies (magnetic resonance imaging and computed tomography) were reviewed. Histopathological subtyping was extracted from the original pathology reports and reflects the classification terminology used at the time of diagnosis (predominantly pre-2022 WHO adenoma terminology). Ki-67 proliferation index was not available in a standardized manner across the entire study period and was therefore not included in the statistical analyses. Clinical parameters, including laboratory and endocrinological data, visual acuity, and formal visual field testing, were evaluated according to the standardized protocol implemented in our department. Postoperative serum cortisol was obtained using a standardized protocol as a fasting morning blood draw at 08:00 a.m. on postoperative day 6. The institutional reference range for morning serum cortisol is 10 - 25 μg/dL. These assessments were performed routinely immediately before and after surgery, as well as at the first follow-up visit, 6–12 weeks postoperatively. Extent of resection (GTR vs STR) and postoperative baseline status were assessed on the first standardized postoperative MRI obtained 6–12 weeks after surgery. This examination was used as the baseline for postoperative volumetric analyses. The selected time window allowed for early standardized assessment while minimizing the influence of immediate postoperative changes associated with very early imaging. MRI acquisition followed a dedicated sellar protocol, including contrast-enhanced T1-weighted sequences and thin-slice T2-weighted imaging. Radiological GTR and recurrence status were derived from the final clinical assessment following routine interpretation of the postoperative reference and serial follow-up MRI examinations by experienced neuroradiologists. The resulting classifications were reviewed longitudinally in the institutional interdisciplinary pituitary tumor board, which included multiple neurosurgeons and neuroradiologists. Thus, endpoint classification reflected a multiprofessional clinical consensus and did not rely on the assessment of a single study reader. To explore preoperative predictors of resection status, all patients with radiological GTR, irrespective of subsequent recurrence, were compared with patients with STR. Continuous and ordinal variables were analyzed using Mann–Whitney U tests. Categorical variables were compared using chi-squared tests with Yates' continuity correction for 2 × 2 contingency tables or Fisher's exact tests, as appropriate. Univariable logistic regression was performed for each candidate predictor. A forced-entry multivariable logistic regression model with STR as the outcome included age, sex, preoperative tumor volume, Hardy grade, Knosp grade, suprasellar extension, and cavernous sinus invasion. Odds ratios (ORs) with 95% confidence intervals (CIs) are reported. Model discrimination was summarized by the apparent area under the receiver operating characteristic curve. If postoperative findings remained stable, subsequent follow-ups were scheduled annually, with deviations from this schedule made on a case-by-case basis following interdisciplinary discussion.

Demographic information, including age and sex, was extracted from the medical records. Surgical variables encompassed the use of microscopic versus endoscopic techniques, duration of surgery, and the intraoperative occurrence of cerebrospinal fluid leakage.

Radiological gross total resection was defined as the absence of identifiable residual tumor on the first standardized postoperative MRI obtained 6–12 weeks after surgery. Recurrence was defined as newly detectable tumor tissue on subsequent follow-up MRI that had not been visible on the postoperative reference examination. Any equivocal tissue on the postoperative reference MRI that could potentially represent residual tumor was conservatively classified as residual disease and therefore as STR rather than GTR. The postoperative development of diabetes insipidus was also systematically documented.

Statistical analyses were performed using MATLAB (MATLAB version: 24.1.0.2537033 (R2024a), Natick, Massachusetts: The MathWorks Inc.; 2024). Missing data were handled by complete-case analysis for the respective variables. Patients with unavailable volumetric data were excluded only from volumetric analyses.

Tumor volume and tumor extension were assessed using established grading systems. Cavernous sinus invasion was evaluated according to the Knosp classification, and tumor invasiveness through the sellar floor was categorized using the Wilson–Hardy system. Pre- and postoperative tumor volumes were manually segmented slice-by-slice with iPlan Net (iPlan Net Cranial 3.0, Brainlab AG, Munich) by a single reader (blinded to outcome, if applicable). In a subset of patients who were initially evaluated with externally acquired imaging of insufficient resolution, tumor volumes could not be reliably segmented; these cases were treated as missing data and excluded from volumetric analyses.

The study was conducted in accordance with the Declaration of Helsinki. Ethical approval was obtained from the local ethics committee of the Technical University of Munich (Ethikkommission der Technischen Universität München; reference number 230/20-S). Owing to the retrospective study design, the requirement for informed consent was waived in accordance with institutional and national regulations.

## 3 Results

### 3.1 Patient characteristics

**Fig. 1:**
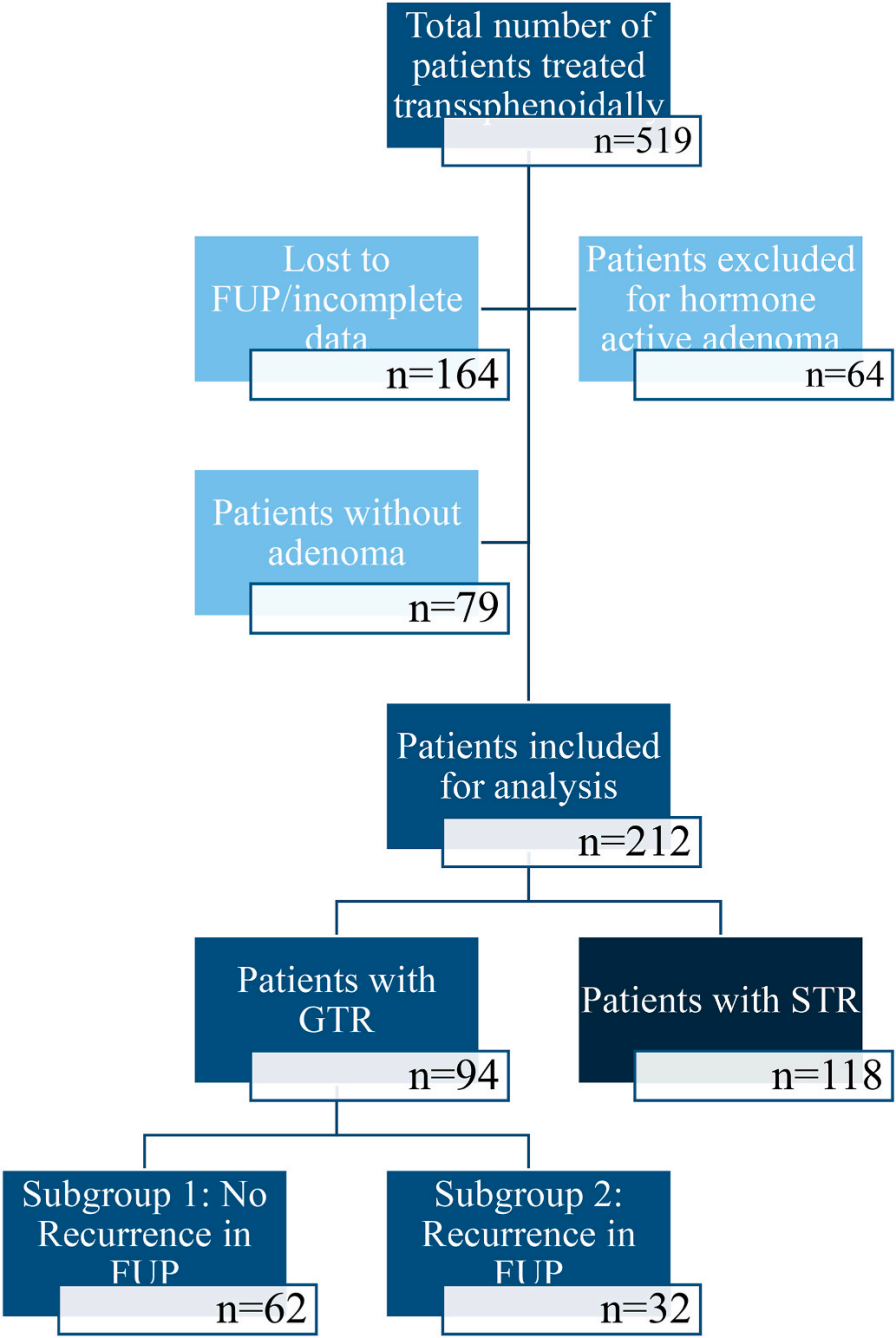
Flowchart describing the number of patients with GTR after the first surgery performed. The no-recurrence subgroup consists of patients who did not experience recurrence during follow-up, whereas in the recurrence subgroup, new tumor tissue was detected in imaging during follow-up examinations. Patients in whom only a subtotal resection was achieved were excluded from the primary recurrence analysis but were included in the secondary exploratory analysis of preoperative predictors of resection status.

We included 212 patients from a total of 519 individuals who underwent surgery for non-functioning pituitary adenomas between 2007 and 2023 at our neurosurgical department. All included patients were treated via MRI-guided transnasal, transsphenoidal microsurgery or an endoscopic endonasal approach and had histopathologically confirmed non-functioning pituitary adenomas. In 94 patients, GTR was confirmed by the postoperative MRI. Among them, 62 patients (66.0%) had no recurrence following gross total resection (GTR) and therefore required only a single surgical procedure during the observation period (no-recurrence subgroup, see Fig. 1). Another group of 32 patients (34.0%) comprises patients with recurrences in whom new tumor tissue was detected in the follow-ups after the initial GTR (recurrence subgroup). These two groups will be analyzed in the following sections with regard to statistical differences. In 118 patients (55.7%), only a partial resection of the tumor was achieved according to postoperative imaging. These patients were excluded from the primary recurrence analysis but were included in the secondary exploratory analysis of preoperative predictors of resection status (see Fig. 1 and Supplementary Table S1 and S2).

Follow-ups were carried out a median of two years and a mean of three years after the first operation.

The overall mean age was 55.10 years (range, 24–85 years). A total of 57 patients (60.6%) were male and 37 (39.4%) were female. Patients in the recurrence subgroup were significantly younger than those in the no-recurrence subgroup (mean age, 51.2 vs. 57.1 years; Mann–Whitney *U* test, p = 0.008). Sex distribution did not differ significantly between the subgroups (53.1% vs. 64.5% male; chi-squared test with Yates’ continuity correction, p = 0.396) (Table 1).

**Table 1:** Patient population and tumor characteristics.

	No-recurrence Subgroup: GTR, no Recurrence	Recurrence subgroup: GTR, Recurrence in FUP	p-value
**Age [years]**	Mean: 57.1	Mean: 51.2	Mann–Whitney U, p = 0.008
	Range: 25 - 85	Range: 24 - 81	
**Sex [absolute M/F (%)]**	40 (64.5%)/22 (35.5%)	17 (53.1%)/15 (46.9%)	Chi-squared, p = 0.396
**Hardy Classification [absolute (%)]**	I: 13 (21.0%)	I: 0 (0%)	Mann–Whitney U, p < 0.001
	II: 30 (48.3%)	II: 13 (40.6%)	
	III: 6 (9.7%)	III: 4 (12.5%)	
	IV: 13(21.0%)	IV: 15 (46.9%)	
**Knosp Classification [absolute (%)]**	0: 1 (1.6%)	0: 0 (0%)	Mann–Whitney U, p = 0.009
	1: 21 (33.9%)	1: 3 (9.4%)	
	2: 17 (27.4%)	2: 12 (37.5%)	
	3: 23 (37.1%)	3: 13 (40.6%)	
	4: 0 (0%)	4: 4 (12.5%)	
**Suprasellar Expansion [absolute (%)]**	40 (64.5%)	29 (90.6%)	Chi-squared, p = 0.014
**Invasion of CS [absolute (%)]**	24 (38.7%)	16 (50.0%)	Chi-squared, p = 0.407
**Operation Method: Endoscopic/Microscopic [absolute (%)]**	12/50 (19.4%/80.6%)	6/26 (18.8%/81.2%)	Chi-squared, p = 1.000
**Tumor Volume preop [cm^3^]**	Mean: 5.54	Mean: 10.45	Mann–Whitney U, p = 0.022
	Range: 0.60 – 37.20	Range: 1.29 – 44.3	
**Histopathology [absolute (%)]**			
• **Null cell**	32 (51.6%)	16 (50.0%)	Chi-squared, p = 0.178
• **LH**	4 (6.5%)	6 (18.8%)	
• **FSH**	2 (3.2%)	2 (6.2%)	
• **LH + FSH**	16 (25.8%)	3 (9.4%)	
• **ACTH**	4 (6.5%)	4 (12.5%)	
• **plurihormonal**	4 (6.5%)	1 (3.1%)	
**Visual Field Deficit [absolute (%)]**	16 (25.8%)	10 (31.3%)	Chi-squared, p = 0.752

### 3.2 Surgical performance

In the no-recurrence subgroup, 12 patients (19.4%) underwent endoscopic surgery, whereas 50 patients (80.6%) were treated microsurgically. In the recurrence subgroup, 6 patients (18.8%) underwent endoscopic surgery, and 26 patients (81.2%) underwent microsurgical surgery. The statistical comparison revealed no significant difference in surgical approach between the two groups (chi-squared test; p = 1.000).

Mean surgery duration in the overall cohort was 60.3 min [20–186 min]. In the no-recurrence subgroup, operative time averaged 59.8 min [24–133 min], whereas procedures in the recurrence subgroup were slightly longer, with a mean duration of 71.5 min [25–120 min]. In the statistical comparison, however, this difference did not reach significance (Mann–Whitney *U* test; p = 0.108).

In the total cohort of 212 patients, gross total resection was achieved in 44.3% (n = 94). Intraoperative cerebrospinal fluid leakage occurred in 64 cases (30.2%), without significant differences between the subgroups (chi-squared test; p = 0.403). Immediate postoperative revision surgery was required in eight patients (4.4%), including two cases of postoperative hemorrhage and six cases of persistent rhinoliquorrhea in the context of intraoperative CSF leakage.

### 3.3 Tumor configuration: Hardy and Knosp classification, suprasellar expansion and invasion of cavernous sinus

**Fig. 2:**
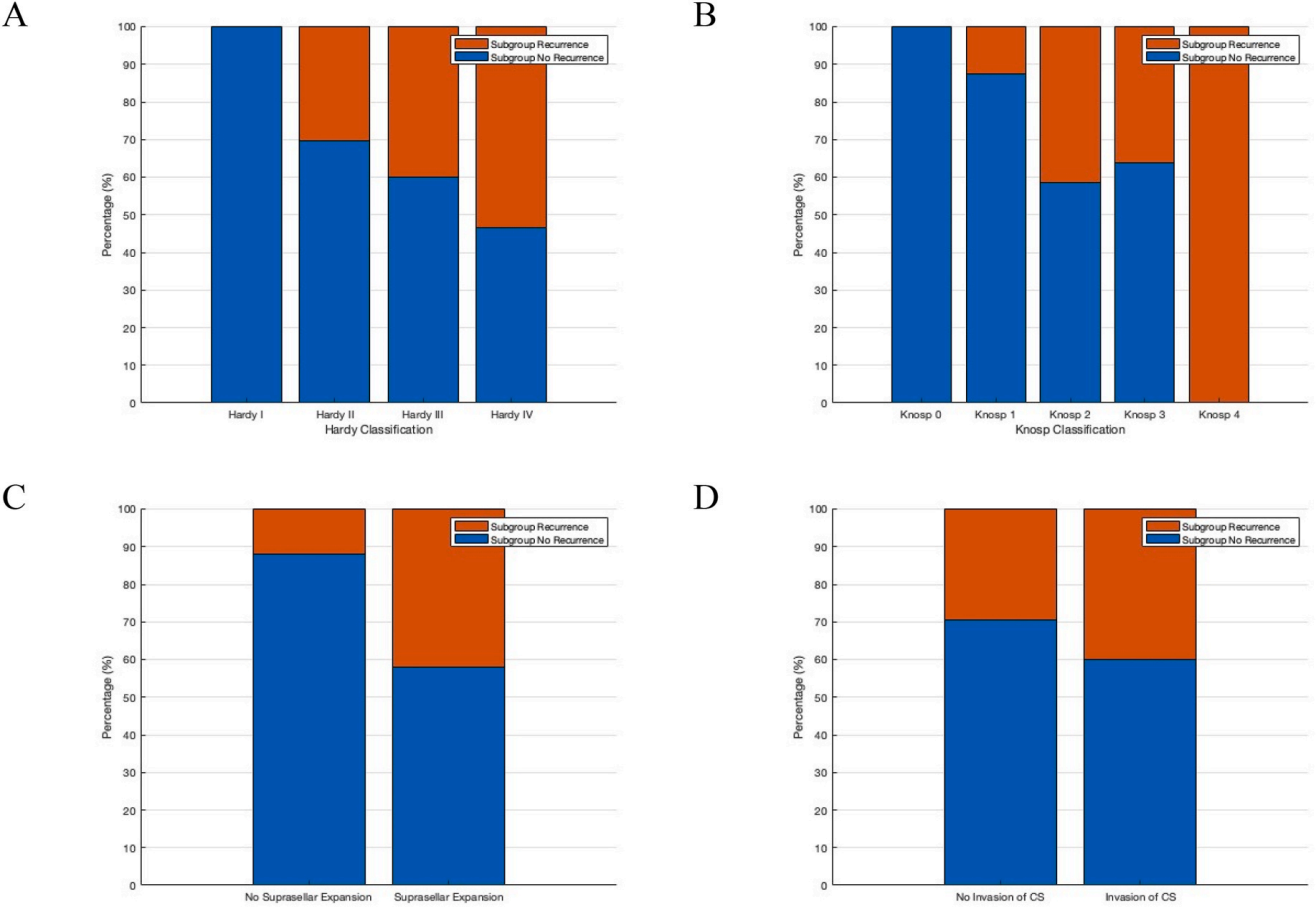
3D configuration of tumors in the no-recurrence and the recurrence subgroup. All graphs represent 100% stacked bar charts, illustrating the relative distribution of each classification within the two subgroups. (A) Distribution of Hardy grades (1–4). (B) Relative distribution of Knosp classification. (C) Proportion of tumors with suprasellar expansion. (D) Frequency of cavernous sinus invasion.

We then analyzed tumor configuration using established radiological classifications (see Table 1). According to the Hardy–Wilson system, the no-recurrence subgroup included 13 grade I tumors (21.0%), 30 grade II tumors (48.3%), 6 grade III tumors (9.7%), and 13 grade IV tumors (21.0%). In the recurrence subgroup, no tumors were classified as grade I (0%), while 13 were grade II (40.6%), 4 were grade III (12.5%), and 15 were grade IV (46.9%) (see Fig. 2A). A Mann–Whitney *U* test demonstrated that patients in the recurrence subgroup exhibited significantly higher Hardy grades than those in the no-recurrence subgroup (U = 603.5, p < 0.001), indicating that lower Hardy grades (I–II) are predominantly associated with no recurrence, whereas higher grades (III–IV) occur more frequently in the recurrence subgroup. A similar pattern was observed for the Knosp classification (see Fig. 2B). In the no-recurrence subgroup, the distribution was Knosp 0: n = 1 (1.6%), Knosp 1: n = 21 (33.9%), Knosp 2: n = 17 (27.4%), Knosp 3: n = 23 (37.1%), and Knosp 4: n = 0 (0%). In the recurrence subgroup, the corresponding distribution was Knosp 0: n = 0 (0%), Knosp 1: n = 3 (9.4%), Knosp 2: n = 12 (37.5%), Knosp 3: n = 13 (40.6%), and Knosp 4: n = 4 (12.5%). A Mann–Whitney *U* test again showed significantly higher Knosp grades in the recurrence subgroup compared with the no-recurrence subgroup (U = 679.0, p = 0.009), reflecting a greater tendency toward cavernous sinus invasion in the recurrence subgroup, whereas lower Knosp grades were more characteristic of the no-recurrence subgroup.

A similar trend was observed in the distribution of suprasellar expansion between the subgroups (see Table 1 and Fig. 2C). Suprasellar expansion was present in 64.5% of cases in the no-recurrence subgroup (40 out of 62) and in 90.6% of cases in the recurrence subgroup (29 out of 32). In contrast, the absence of suprasellar expansion was more frequent in the no-recurrence (35.5%) than in recurrence subgroup (9.4%). A chi-square test confirmed a statistically significant association between suprasellar expansion and subgroup affiliation (χ^2^(1) = 6.09, p = 0.014), showing that suprasellar extension was more frequent in the recurrence subgroup than in the no-recurrence subgroup. Following the analysis of Hardy and Knosp grades and suprasellar extension, cavernous sinus invasion was compared between subgroups (Table 1 and Fig. 2D). Cavernous sinus invasion was present in 24 of 62 patients (38.7%) in the no-recurrence subgroup and in 16 of 32 patients (50.0%) in the recurrence subgroup. This difference was not statistically significant (χ^2^(1) = 0.687, p = 0.407).

### 3.4 Tumor volume and histological subtype

**Fig. 3:**
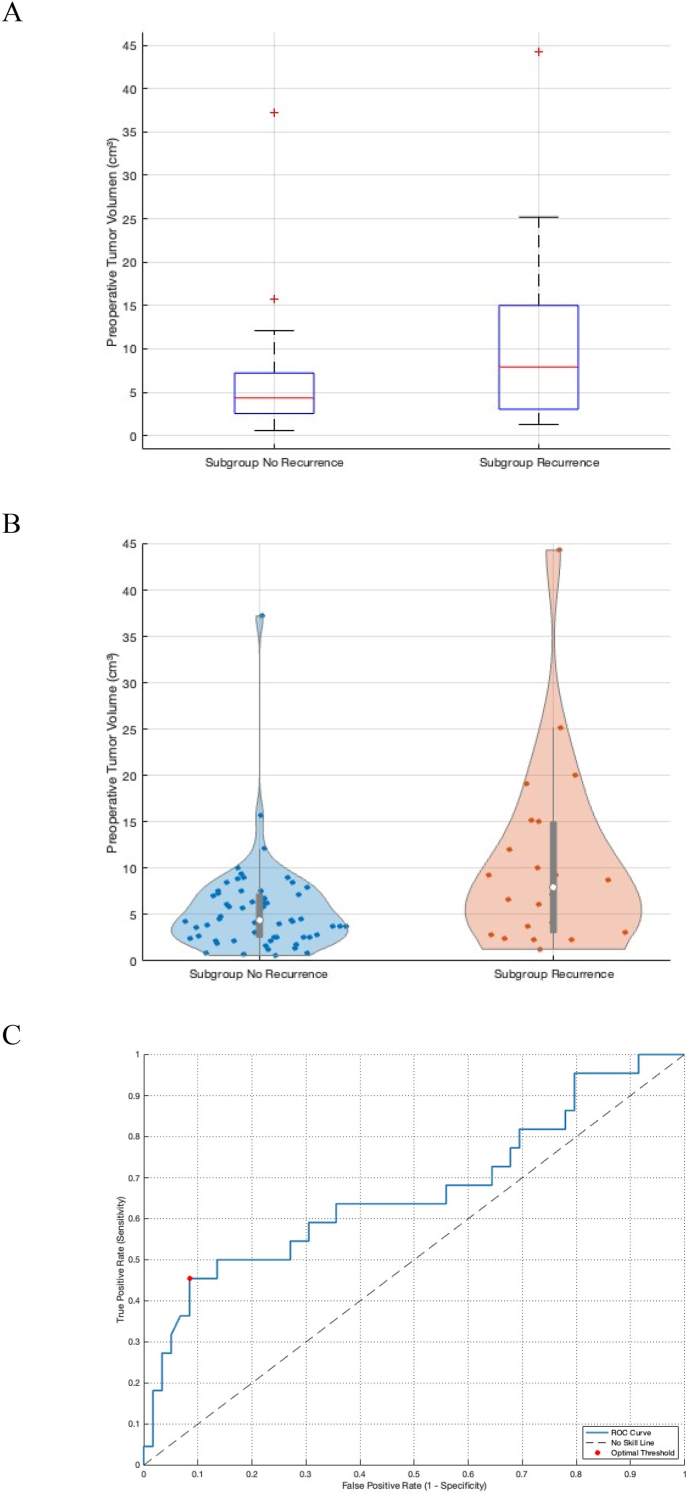
Comparison of Preoperative Tumor Volume and ROC Analysis for Subgroup Classification. (A) Boxplot depicting the distribution of preoperative tumor volume in the no-recurrence subgroup and the recurrence subgroup. (B) Violin plot illustrating the density distribution of preoperative tumor volume for both subgroups. (C) ROC curve assessing the discriminative power of tumor volume for subgroup classification, with the optimal cutoff value (9.22 cm^3^) marked by a red dot.

Another characteristic that defines the tumor, in addition to those mentioned above, is the tumor volume, which can be estimated using 3D MRI imaging. Preoperative tumor volume differed between the no-recurrence and the recurrence subgroup (see Table 1). In patients without recurrence, the mean tumor volume was 5.54 cm^3^ (median: 4.35 cm^3^, range: 0.6–37.2 cm^3^) with a standard deviation of 5.19 cm^3^. In patients with recurrence, tumors were larger on average, with a mean volume of 10.45 cm^3^ (median: 7.93 cm^3^, range: 1.29–44.3 cm^3^) and a standard deviation of 10.01 cm^3^ (Fig. 3A + B). A Mann-Whitney *U* test confirmed a statistically significant difference in preoperative tumor volume between the two subgroups (U = 432.5, p = 0.022), indicating that tumors in the recurrence subgruop were significantly larger compared to those in the no-recurrence subgroup.

To assess the ability of preoperative tumor volume to distinguish between the no-recurrence and the recurrence subgroup, a Receiver Operating Characteristic (ROC) curve analysis was performed (Fig. 3C). The area under the curve (AUC) was 0.667, indicating a moderate discriminative power.

Using the Youden Index, an optimal cutoff value of 9.22 cm^3^ was determined, which yielded a sensitivity of 45.5% and a specificity of 91.5%. This suggests that while tumor volume alone is not a perfect classifier, larger tumor volumes are more indicative of the recurrence subgroup, with a high specificity but relatively low sensitivity.

The distribution of histological subtypes was analyzed between the no-recurrence and recurrence subgroups. The most common subtype was null cell adenoma, occurring in 32 cases (51.6%) in the no-recurrence subgroup and 16 cases (50.0%) in the recurrence subgroup. The remaining subtypes in the no-recurrence and recurrence subgroups, respectively, were LH (4 [6.5%] vs. 6 [18.8%]), FSH (2 [3.2%] vs. 2 [6.2%]), LH + FSH (16 [25.8%] vs. 3 [9.4%]), ACTH (4 [6.5%] vs. 4 [12.5%]), and plurihormonal adenomas (4 [6.5%] vs. 1 [3.1%]). A chi-squared test did not reveal a statistically significant association between histological subtype and subgroup affiliation (χ^2^(5) = 7.63, p = 0.178.

### 3.5 Clinical features: hormone levels and visual impairment

For another analysis, cortisol levels measured immediately before and five days after surgery, as part of standard clinical practice, were evaluated.

Preoperative cortisol levels showed no significant difference between the no-recurrence and the recurrence subgroup. In the cohort of patients without recurrence in FUP, the mean cortisol level was 13.29 μg/dl, with a standard deviation of 12.77 μg/dl. In the recurrence subgroup, cortisol levels were slightly lower, with a mean of 11.10 μg/dl and a standard deviation of 6.96 μg/dl. The Mann-Whitney *U* test indicated no statistically significant difference between the groups (U = 1140.000, p = 0.540).

Postoperative cortisol levels remained similar between the two groups. In the no-recurrence subgroup, the mean cortisol level was 12.12 μg/dl, with a standard deviation of 8.46 μg/dl. In the recurrence-subgroup, the mean postoperative cortisol level was 11.84 μg/dl with a standard deviation of 7.87 μg/dl. Similar to the preoperative values, the Mann-Whitney *U* test did not show a statistically significant difference between the groups (U = 1139.000, p = 0.871).

**Fig. 4:**
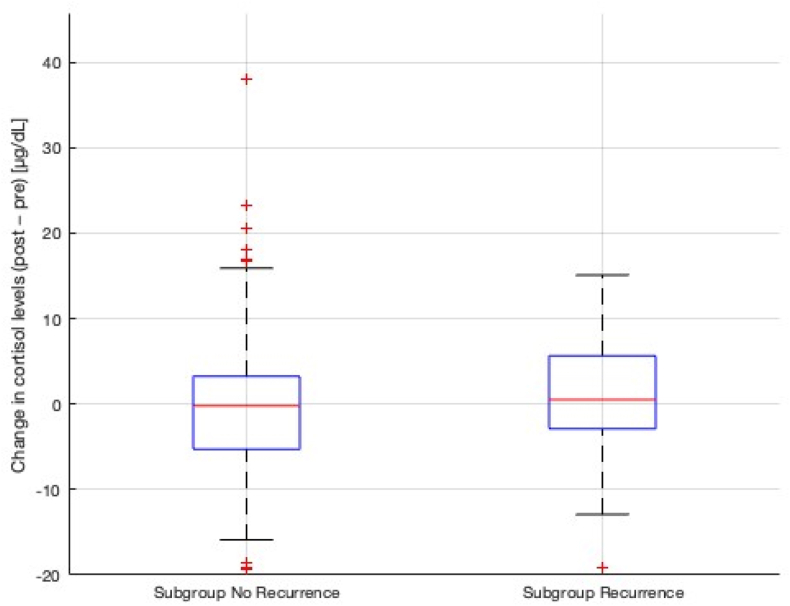
Box plot illustrating the distribution of perioperative cortisol changes (postoperative minus preoperative) in the no-recurrence and recurrence subgroups. The median and interquartile range are displayed, with whiskers representing 1.5 times the interquartile range. Outliers are shown as individual points. The between-group difference was statistically significant (p = 0.035).

When comparing pre-to postoperative cortisol levels per patient, different trends were observed in the two subgroups (see Fig. 4). In the no-recurrence subgroup, cortisol levels decreased on average by 1.17 μg/dl, whereas in the recurrence subgroup, they increased slightly, with a mean change of +0.50 μg/dl. A Mann–Whitney *U* test revealed a statistically significant difference in the change in cortisol levels between the two subgroups (U = 300.000, p = 0.035), yet the small absolute difference limits its clinical interpretability.

Another preoperative clinical factor analyzed in our cohort was the specific visual field defect, particularly bitemporal hemianopia. Visual field deficits were present in 16 out of 62 patients (25.8%) in the no-recurrence subgroup, while 46 patients (74.2%) had no impairment. In the recurrence subgroup, 10 out of 32 patients (31.3%) exhibited a visual field deficit, whereas 68.8% had no deficits. A chi-square test revealed no statistically significant difference in the distribution of visual field deficits between the two subgroups (χ^2^(1) = 0.10, p = 0.752).

### 3.6 Exploratory analysis of preoperative predictors of resection status

To explore preoperative characteristics associated with radiological GTR, all 94 patients with GTR were compared with the 118 patients with STR. Patients with STR were older than patients with GTR (median 59.0 vs. 55.5 years; p = 0.017) and had larger preoperative tumor volumes (median 7.37 vs. 4.74 cm^3^; p < 0.001). Higher Hardy and Knosp grades were associated with STR (p = 0.008 and p < 0.001, respectively), and suprasellar extension was more frequent in the STR group (87.3% vs. 73.4%; p = 0.010). Cavernous sinus invasion was also more frequent in the STR group (55.1% vs. 42.6%), although this difference did not reach statistical significance (p = 0.070).

An exploratory forced-entry multivariable logistic regression model included age, sex, preoperative tumor volume, Hardy grade, Knosp grade, suprasellar extension, and cavernous sinus invasion. Complete data were available for 191 patients. Higher Knosp grade (OR 1.81 per one-grade increase, 95% CI 1.05–3.09; p = 0.031) and increasing age (OR 1.025 per year, 95% CI 1.001–1.049; p = 0.040) remained associated with STR. Preoperative tumor volume was not independently associated with STR after adjustment for the correlated anatomical variables (OR 1.04 per cm^3^, 95% CI 0.98–1.10; p = 0.169). The apparent model AUC was 0.708. Full univariable and multivariable results are provided in Supplementary Table S1 and S2.

## 4 Discussion

Our data indicate that preoperative tumor burden and radiological configuration are associated with recurrence after radiological GTR. Patients in the recurrence subgroup had larger preoperative tumor volumes and higher Hardy–Wilson and Knosp grades, and suprasellar extension was more frequent. These findings should not be interpreted as demonstrating an independent causal effect of suprasellar extension. Rather, suprasellar extension may serve as a marker of greater tumor burden and three-dimensional anatomical complexity.

From an anatomical perspective, irregular or lobulated tumor portions extending through the diaphragmatic aperture may be closely related or adherent to the diaphragma sellae, arachnoid membranes, pituitary stalk, optic apparatus, and adjacent vessels. These relationships can restrict direct visualization and safe surgical manipulation of superior tumor components and may increase the likelihood that very small peripheral or adherent remnants remain below the detection threshold of postoperative MRI. Suprasellar extension may also increase the likelihood of arachnoid opening and intraoperative cerebrospinal fluid leakage. However, intraoperative cerebrospinal fluid leakage was not associated with recurrence in our cohort, and our data therefore do not support cerebrospinal fluid leakage as a mediator of the observed association.

The apparent difference from studies of residual tumor behavior after STR may be endpoint-specific. Once a visible residual tumor is present, subsequent progression may depend more strongly on residual volume and tumor-intrinsic growth kinetics. By contrast, recurrence after apparent GTR may partly reflect delayed detection of MRI-occult residual tumor tissue. In our previously published analysis of the same overall institutional cohort, suprasellar extension did not distinguish stable from progressive residual tumors after STR (Eisenkolb et al., 2026). Although radiological GTR was documented in all cases in the present study, anatomical complexity may therefore influence recurrence through limitations of resectability rather than through a direct effect on growth. Spatial biological heterogeneity may provide an additional explanation: the reported greater invasive capability of peripheral regions of pituitary macroadenomas supports the possibility that microscopic invasive extensions can extend beyond the macroscopically or radiologically apparent tumor boundary (Lee et al., 2025). This observation does not establish the mechanism in our cohort but provides a plausible biological complement to the anatomical explanation. Prior work has similarly shown that anatomical invasion rather than surgical technique alone may limit the feasibility of complete resection (Pei et al., 2022; Micko et al., 2015; Chang et al., 2025). Differences between studies regarding the prognostic value of Hardy and Knosp classifications after GTR may relate to variability in imaging resolution, surgical approach, or GTR definitions, or may indicate that some anatomical patterns denote more infiltrative tumors (Shen et al., 2024; Fang et al., 2021; Araujo-Castro et al., 2021; Lu et al., 2022). Taken together, our findings support incorporating preoperative volumetry and tumor configuration (suprasellar expansion, cavernous sinus invasion, Hardy/Knosp grade) into postoperative risk stratification to identify patients who warrant closer surveillance or tailored follow-up, while prospective multicenter validation is required.

Several prior studies have reported conflicting findings regarding the prognostic role of histological subtype after radiological GTR. In line with our results, some investigations have found no independent association between specific adenoma subtypes and recurrence once extent of resection and anatomical factors are accounted for (Fountas et al., 2018; Gupta et al., 2024; McClure et al., 2024), whereas other reports, particularly focusing on silent corticotroph adenoma, have described an increased recurrence risk, although not consistently replicated (Goyal-Honavar et al., 2021; Langlois et al., 2018). These discrepant findings may reflect small sample sizes for rare subtypes, varying histopathological and immunohistochemical classification criteria over time, differences in follow-up duration, and the absence of standardized molecular profiling across cohorts. Consequently, the lack of an observable effect in our series does not exclude a true biological contribution of certain subtypes but instead highlights the need for larger multicenter studies with uniform histopathological and molecular characterization to resolve these inconsistencies.

A noteworthy and potentially clinically meaningful finding in our study was the difference in postoperative cortisol changes between the two subgroups. Patients without recurrence exhibited a modest postoperative decrease, whereas those that presented with recurrence in FUP showed a slight increase. When interpreting these findings, it is important to consider that postoperative cortisol trajectories show substantial variability in the literature: cortisol levels may decrease, remain stable or rise (Mayberg et al., 2018; Butenschoen et al., 2022; Zhao et al., 2023). The smaller cortisol decline in the recurrence subgroup could reflect less surgical manipulation of normal pituitary tissue in critical regions, potentially allowing microscopic remnants to remain despite radiological GTR. Another possible explanation is that the differing cortisol trajectories reflect variations in pituitary-adrenal axis reserve or feedback mechanisms that are independent of surgical factors. Because early postoperative cortisol measurements have limited predictive value for long-term adrenal function or recurrence risk (Mayberg et al., 2018; Butenschoen et al., 2022), and measurements at 6 to 12 weeks are considered more reliable, our findings should be viewed as hypothesis-generating and require prospective validation using standardized endocrine assessment protocols.

The distribution of surgical approaches was nearly identical between the two subgroups, indicating that recurrence after radiological GTR cannot be attributed solely to differences in operative technique. This suggests that anatomical and tumor-intrinsic factors play a larger role in determining long-term outcomes than whether surgery was performed microscopically or endoscopically. However, the relatively small number of endoscopic procedures in our cohort limits definitive conclusions regarding potential technique-related effects, and larger series with balanced surgical approaches are needed to clarify this aspect.

Preoperative assessment of tumor consistency may provide additional information on resectability and recurrence risk. Firm or fibrotic tumors can be more difficult to debulk and dissect from adjacent neurovascular structures, potentially reducing the probability of complete resection and increasing the likelihood of small residual tumor portions. In the present cohort, intraoperative consistency was not recorded using a standardized grading system, and MRI sequence parameters were not sufficiently uniform across the 2007–2023 study period to permit a reliable retrospective MRI-based assessment. Tumor consistency was therefore not included as an analytical variable. Prospective studies combining standardized MRI markers of consistency with structured intraoperative assessment are warranted.

Preoperative visual field deficits were observed in both subgroups at comparable frequencies, and no statistical association with recurrence could be identified, which is in line with current literature (Subramanian et al., 2021). Visual impairment, therefore, appears to reflect the anatomical relationship between the tumor and the optic pathways rather than intrinsic tumor biology. The severity or presence of visual symptoms is more likely determined by the direction and extent of tumor growth, not by factors that influence the likelihood of tumor regrowth after gross total resection.

The results of this study support a more differentiated approach to postoperative surveillance after radiological GTR. In this cohort, larger preoperative tumor volumes, higher Hardy or Knosp grades, and suprasellar extension were associated with a higher observed recurrence frequency and may justify consideration of shorter MRI intervals and prolonged radiological follow-up. Even though the predictive strength of individual parameters is moderate, their combined assessment enables a more targeted allocation of surveillance resources and may help reduce unnecessary imaging in patients with low-risk tumor characteristics. Integrating these factors into follow-up algorithms could therefore improve efficiency while maintaining patient safety.

The exploratory analysis of resection status showed that larger tumors, higher Hardy and Knosp grades, and suprasellar extension were associated with STR in univariable analyses. After simultaneous adjustment, Knosp grade and age remained associated with STR, whereas preoperative tumor volume did not. This suggests that the unadjusted association between tumor volume and resectability may be partly captured by correlated measures of parasellar and sellar invasion. These findings should be interpreted as secondary and hypothesis-generating because resection status was not the primary endpoint and the model was not externally validated.

Several methodological limitations should be considered when interpreting the results of this study. First, the retrospective design inherently carries a risk of selection bias, variability in diagnostic work-up, and differences in follow-up intensity across patients. Second, the study spans a 16-year period during which relevant clinical standards evolved, including improvements in MRI resolution, refinements in surgical techniques, and changes in neuropathological classification criteria. These temporal effects may introduce heterogeneity that cannot be fully controlled. Furthermore, tumor consistency was not systematically documented and could not be reconstructed reliably from heterogeneous MRI protocols, representing a potential source of residual confounding. Third, tumor volumes were obtained through manual segmentation by a single reader, and interobserver reproducibility of the volumetric measurements was therefore not assessed. GTR and recurrence status, by contrast, reflected routine neuroradiological reporting and longitudinal multidisciplinary review rather than a single study reading. Although this multiprofessional consensus process reduces the likelihood of clinically relevant misclassification, it was not a dedicated blinded central rereading and did not provide a formal measure of interobserver agreement. Observer-dependent classification therefore cannot be completely excluded. Moreover, clinical imaging consensus cannot exclude microscopic or MRI-occult residual tumor tissue. Fourth, only 94 of 212 patients were classified as having achieved gross total resection. This relatively low proportion likely reflects our strictly MRI-based definition of GTR, as even minimal or equivocal postoperative findings that could represent either scarring or small tumor remnants were conservatively categorized as subtotal resection. This approach increases the specificity of the GTR cohort but may limit generalizability to settings that use less stringent imaging criteria. Finally, the number of patients with recurrence was relatively small (n = 32), which restricts statistical power and limits the robustness of subgroup and exploratory analyses. Larger prospective studies are therefore needed to validate the identified predictors.

Future research should aim to validate the proposed volumetric cutoffs in prospective, uniformly assessed patient cohorts to confirm their clinical applicability. Integrating molecular and proliferative markers—such as Ki-67, PIT-1, or TPIT expression—may further refine risk stratification and help differentiate tumors with distinct biological behaviors. Advances in radiomics and machine learning offer additional opportunities to develop predictive models based on preoperative imaging features, potentially enabling individualized recurrence risk assessment before surgery. Furthermore, analyzing tumor growth kinetics over time, rather than relying solely on binary recurrence definitions, may provide a more nuanced understanding of postoperative tumor behavior and facilitate tailored follow-up strategies.

## 5 Conclusion

Preoperative tumor volume and radiological markers of tumor configuration, including Hardy and Knosp grades as well as suprasellar extension, were associated with recurrence after radiological gross total resection. These findings suggest that anatomical complexity and tumor burden may provide clinically relevant prognostic information in addition to postoperative imaging. Incorporating these parameters into postoperative surveillance may support more tailored follow-up strategies. Prospective studies are warranted to validate these results and to refine risk stratification using molecular and advanced imaging approaches.

## Funding

This research did not receive any specific grant from funding agencies in the public, commercial, or not-for-profit sectors.

## Declaration of competing interest

The authors declare that they have no known competing financial interests or personal relationships that could have appeared to influence the work reported in this paper.
