# Supplementary material for: Preoperative risk stratification for recurrence after gross total resection of non-functioning pituitary macroadenomas: The role of tumor volume and 3D anatomical complexity — A retrospective single-center cohort study
Source: Brain Spine. 2026 Aug 7;6:106277. doi: 10.1016/j.bas.2026.106277 (PMC13485606; doi:10.1016/j.bas.2026.106277)
Supplement: Supplementary file 1 [file mmc1.docx]

| **Variable** | **GTR** | **STR** | **p-value** |
| --- | --- | --- | --- |
| Age, years | 55.5 [43.0–67.8] (n=94) | 59.0 [50.3–71.0] (n=118) | 0.017 |
| Male sex, n/N (%) | 57/94 (60.6%) | 71/118 (60.2%) | 0.945 |
| Preoperative tumor volume, cm³ | 4.74 [2.62–8.41] (n=81) | 7.37 [4.57–15.23] (n=110) | <0.001 |
| **Hardy grade, n/N (%)** |  |  | **0.008** |
| Grade I | 13/94 (13.8%) | 3/113 (2.7%) |  |
| Grade II | 43/94 (45.7%) | 42/113 (37.2%) |  |
| Grade III | 10/94 (10.6%) | 28/113 (24.8%) |  |
| Grade IV | 28/94 (29.8%) | 40/113 (35.4%) |  |
| **Knosp grade, n/N (%)** |  |  | **<0.001** |
| Grade 0 | 1/94 (1.1%) | 0/114 (0.0%) |  |
| Grade 1 | 24/94 (25.5%) | 5/114 (4.4%) |  |
| Grade 2 | 29/94 (30.9%) | 46/114 (40.4%) |  |
| Grade 3 | 36/94 (38.3%) | 37/114 (32.5%) |  |
| Grade 4 | 4/94 (4.3%) | 26/114 (22.8%) |  |
| Suprasellar extension, n/N (%) | 69/94 (73.4%) | 103/118 (87.3%) | 0.010 |
| Cavernous sinus invasion, n/N (%) | 40/94 (42.6%) | 65/118 (55.1%) | 0.070 |
| Preoperative visual-field deficit, n/N (%) | 26/80 (32.5%) | 46/107 (43.0%) | 0.145 |

Supplementary Table S1: Preoperative characteristics according to radiological extent of resection. Values are median [interquartile range] unless otherwise stated. P-values were calculated using Mann–Whitney U tests for continuous and ordinal variables and Pearson chi-squared tests for categorical variables. Analyses used available cases; denominators are shown where data were missing. GTR, gross total resection; STR, subtotal resection.
