# Supplementary material for: Preoperative risk stratification for recurrence after gross total resection of non-functioning pituitary macroadenomas: The role of tumor volume and 3D anatomical complexity — A retrospective single-center cohort study
Source: Brain Spine. 2026 Aug 7;6:106277. doi: 10.1016/j.bas.2026.106277 (PMC13485606; doi:10.1016/j.bas.2026.106277)
Supplement: Supplementary file 2 [file mmc2.docx]

| **Predictor** | **Scale / reference** | **n** | **Univariable OR (95% CI)** | **p-value** | **Multivariable OR (95% CI)** | **p-value** |
| --- | --- | --- | --- | --- | --- | --- |
| Age | Per year | 212 | 1.025 (1.005–1.045) | 0.012 | 1.025 (1.001–1.049) | 0.040 |
| Sex | Male vs female | 212 | 0.981 (0.563–1.707) | 0.945 | 0.899 (0.464–1.745) | 0.754 |
| Preoperative tumor volume | Per 1 cm³ | 191 | 1.073 (1.025–1.124) | 0.003 | 1.039 (0.984–1.096) | 0.169 |
| Hardy grade | Per one-grade increase | 207 | 1.456 (1.097–1.932) | 0.009 | 1.027 (0.699–1.511) | 0.891 |
| Knosp grade | Per one-grade increase | 208 | 1.998 (1.436–2.782) | <0.001 | 1.805 (1.054–3.091) | 0.031 |
| Suprasellar extension | Present vs absent | 212 | 2.488 (1.224–5.056) | 0.012 | 1.880 (0.745–4.744) | 0.182 |
| Cavernous sinus invasion | Present vs absent | 212 | 1.656 (0.958–2.860) | 0.071 | 0.746 (0.328–1.697) | 0.485 |
| Preoperative visual-field deficit | Present vs absent | 187 | 1.566 (0.856–2.867) | 0.146 | Not included | — |

Supplementary Table S2: Univariable and multivariable logistic regression of preoperative predictors of STR**.** Outcome: STR = 1. GTR n = 94; STR n = 118. Univariable models used available cases for each predictor. The forced-entry multivariable model included age, sex, preoperative tumor volume, Hardy grade, Knosp grade, suprasellar extension, and cavernous sinus invasion and included 191 complete cases (81 GTR, 110 STR). The apparent model AUC was 0.708. Odds ratios greater than 1 indicate higher odds of STR. The analysis is exploratory; resection status was not the primary endpoint, and the model was not externally validated.
